# A New Doctrine about Drunkenness and Responsibility

**Published:** 1890-09

**Authors:** 


### A NEW DOCTRINE ABOUT DRUNKENNESS AND RESPONSIBILITY.

---

Another ancient dogma bids fair to become obsolete; we mean the doctrine asserting the responsibility of the drunken person for the criminal acts done in the condition of drunkenness. It was at least as early as the time of Chief Justice Coke that the legal dictum regarding the double guilt of the inebriate criminal was formulated, to the effect that inebriety always aggravates the offence, and that the penalty should be increased rather than dimished. This view has generally been entirely acceptable to the legal mind until a recent date, since its tendency was eminently conservative of social and proprietary rights, and *pari passu* entirely acceptable to the general public. But nowadays there is a growing criticism of that theory, and eminent jurists in England and elsewhere have decided that a man may be convicted of an offence committed in drink, and yet be absolved from responsibility; in other words, the criticism has reference not so much to the expediency of the old doctrine—that remains the same, but the justice of the theory is challenged. For example, as reported in the *Lancet*, Baron Pollock has held that the plea of irresponsibility was tenable in a case where a homicide was committed by a person after taking a small quantity of alcoholic liquor—a quantity not sufficient ordinarily to disturb the reasoning faculties, but which, in the case in question, was sufficient to set in motion an insane predisposition that became the prime agent in the manslaughter. Another eminent Judge in England has recently ruled, in regard to the case of a drunken mother, who through the neglect of her babe occasioned its death by starvation, that the withholding of nourishment by the mother was not a crime, for the reason that it was undesigned, and that it is not a declared crime to imbibe too much liquor.

Chief-Baron Pallas has ruled that neither law nor common sense can hold a man responsible for the acts done under the influence of an intoxicant, if by reason of long vigil, deprivation of sleep, or impoverishment of the blood, he shall have become so reduced as to be made drunken with a smaller quantity of liquor than would have produced that effect upon him in good health. Justice Day has gone still further and has declared that a person who does not know the nature and quality of the acts he commits is not responsible for them, whatever may be the cause of his unconsciousness.

The thought contained in these decisions is a complete reversal of the ground of decisions that have been respected for three hundred years by the general public, that have been on a thousand grave occasions accepted by the criminals themselves without a whisper of dissent, and that have passed almost unquestioned by jurists until within the present decade. A recent judicial charge may be quoted to show the tenor that has prevailed in regard to the plea of irresponsibility: "No insanity or irresponsibility can be predicated in any given case unless the mind showed a continuance of delirium or delusion, and that in no case should this be taken into account, in mitigation of guilt, if it resulted from alcoholic intoxication." The ground of precedent, as ordinarily assumed by the State's attorney, whose business it is to bring to punishment as many criminals as possible, is commonly based on old English decisions that intoxication is never an excuse for crime, and that "no man can plead that he should be exempt from the law by reason of not knowing or not being able to control the extent and force of his acts, by reason of being drunk, and that drunkenness is a voluntary insanity, and those who use alcohol to that degree know full well the consequences of that act." These are the extremes of the question as to how far inebriety may coexist with irresponsibility. It is only of late years that there has been enough of practical psychology known by physicians to enable them to grapple with this problem, and still fewer have had that assurance of their convictions to support them against an intrenched and popular doctrine that has ruled the courts for three centuries. The Congress of Psychology, held at Paris during August, 1889, considered this subject and took formal action thereon, according to which it is proposed that the Government should be called upon to take steps to more thoroughly protect society against criminal dipsomaniacs, and for that purpose to establish special asylums for the treatment of habitual drunkenness. This proposition was adopted by the assembly unanimously.—*Gaillard's Medical Journal.*

